# Gender-specific insights into adherence to Mediterranean diet and lifestyle: analysis of 4,000 responses from the MEDIET4ALL project

**DOI:** 10.3389/fnut.2025.1570904

**Published:** 2025-07-29

**Authors:** Mohamed Ali Boujelbane, Achraf Ammar, Atef Salem, Mohamed Kerkeni, Khaled Trabelsi, Bassem Bouaziz, Liwa Masmoudi, Juliane Heydenreich, Christiana Schallhorn, Gabriel Müller, Ayse Merve Uyar, Hadeel Ali Ghazzawi, Adam Tawfiq Amawi, Bekir Erhan Orhan, Giuseppe Grosso, Osama Abdelkarim, Tarak Driss, Kais El Abed, Piotr Zmijewski, Nasreddine Benbettaieb, Clément Poulain, Laura Reyes, Amparo Gamero, Marta Cuenca-Ortolá, Nicola Francesca, Concetta Maria Messina, Björn Lorenzen, Stefania Filice, Aadil Bajoub, El-Mehdi Ajal, El Amine Ajal, Majdouline Obtel, Sadjia Lahiani, Taha Khaldi, Nafaa Souissi, Omar Boukhris, Haitham Jahrami, Waqar Husain, Walid Mahdi, Hamdi Chtourou, Wolfgang I. Schöllhorn

**Affiliations:** ^1^Department of Training and Movement Science, Institute of Sport Science, Johannes Gutenberg-University Mainz, Mainz, Germany; ^2^High Institute of Sport and Physical Education of Sfax, University of Sfax, Sfax, Tunisia; ^3^Research Laboratory, Molecular Bases of Human Pathology, LR19ES13, Faculty of Medicine of Sfax, University of Sfax, Sfax, Tunisia; ^4^Department of Movement Sciences and Sports Training, School of Sport Science, The University of Jordan, Amman, Jordan; ^5^Research Laboratory Education, Motricity, Sport and Health, EM2S, LR19JS01, High Institute of Sport and Physical Education of Sfax, University of Sfax, Sfax, Tunisia; ^6^Multimedia InfoRmation systems and Advanced Computing Laboratory (MIRACL), University of Sfax, Sfax, Tunisia; ^7^Higher Institute of Computer Science and Multimedia of Sfax (ISIMS), University of Sfax, Sfax, Tunisia; ^8^Faculty of Sports Sciences, Department of Experimental Sports Nutrition, Leipzig University, Leipzig, Germany; ^9^Department of Sports Economics, Sociology and History, Institute of Sport Science, Johannes Gutenberg-University Mainz, Mainz, Germany; ^10^Department of Nutrition and Food Technology, School of Agriculture, The University of Jordan, Amman, Jordan; ^11^Faculty of Sports Sciences, Istanbul Aydın University, Istanbul, Türkiye; ^12^Department of Biomedical and Biotechnological Sciences, University of Catania, Catania, Italy; ^13^Faculty of Sport Sciences, Assiut University, Assiut, Egypt; ^14^ESLSCA University Egypt, Giza, Egypt; ^15^Interdisciplinary Laboratory in Neurosciences, Physiology and Psychology Physical Activity, Health and Learning (LINP2), UFR STAPS, Paris Nanterre University, Nanterre, France; ^16^Department of Biomedical Sciences, Jozef Pilsudski University of Physical Education in Warsaw, Warsaw, Poland; ^17^Department BioEngineering, Institut Universitaire de Technologie IUT-Dijon-Auexrre-Nevers, University Burgundy Europe, Dijon, France; ^18^Joint Research Unit UMR PAM-PCAV (Physical-Chemistry of Food and Wine Laboratory), Université Bourgogne Europe/Institut AgroDijon/INRAE, Dijon, France; ^19^Vitagora Innovation Cluster, Dijon, France; ^20^Department of Preventive Medicine and Public Health, Food Science, Toxicology and Forensic Medicine, Faculty of Pharmacy & Food Sciences, University of Valencia, Valencia, Spain; ^21^Department of Agricultural Food and Forest Sciences, University of Palermo, Palermo, Italy; ^22^Laboratory of Marine Biochemistry and Ecotoxicology, Department of Earth and Marine Sciences DiSTeM, University of Palermo, Trapani, Italy; ^23^Microtarians Academy, Luxembourg, Luxembourg; ^24^Laboratory of Food and Food By-Products Chemistry and Processing Technology, National School of Agriculture in Meknès, Meknès, Morocco; ^25^Laboratory of Social Medicine, Faculty of Medicine and Pharmacy of Rabat, Department of Epidemiology and Public Health, Mohammed V University, Rabat, Morocco; ^26^UPR of Pharmacognosy, Faculty of Medicine and Pharmacy of Rabat, Mohammed V University, Rabat, Morocco; ^27^VALCORE Laboratory, Faculty of Science, Department of Biology, University of M’hamed Bougara Boumerdes, Boumerdes, Algeria; ^28^Biotechnology Research Center (C.R.Bt), Constantine, Algeria; ^29^SIESTA Research Group, School of Allied Health, Human Services and Sport, La Trobe University, Melbourne, VIC, Australia; ^30^Sport, Performance, and Nutrition Research Group, School of Allied Health, Human Services and Sport, La Trobe University, Melbourne, VIC, Australia; ^31^Government Hospitals, Manama, Bahrain; ^32^Department of Psychiatry, College of Medicine and Medical Sciences, Arabian Gulf University, Manama, Bahrain; ^33^Department of Humanities, COMSATS University Islamabad, Islamabad, Pakistan

**Keywords:** Mediterranean diet, gender differences, MedLife index, physical activity, mental health

## Abstract

**Background:**

The Mediterranean Diet (MedDiet) is widely recognized for its health benefits, though adherence varies across populations and is influenced by multiple lifestyle and demographic factors. This study examined MedDiet adherence patterns and their associations with lifestyle behaviors, with particular attention to gender differences in a large, multinational cohort.

**Methods:**

Data were obtained via the MEDIET4ALL survey, an international cross-sectional study that included 4,010 participants (mean age: 36.04 ± 15.06 years; 59.5% female) across 10 countries. The evaluation of adherence to the MedDiet was conducted using the MedLife Index, a validated tool that assesses adherence to MedDiet patterns and lifestyle behaviors through three blocks: Mediterranean food consumption, MedDiet habits, and lifestyle behaviors. Additionally, validated instruments were used to measure associated factors, including perceived barriers to adherence, physical activity, sleep quality and disturbances, mental health, life satisfaction, social participation, and technology use behaviors.

**Results:**

While total Mediterranean lifestyle (MedLife) scores showed no significant gender differences, women demonstrated better adherence to food consumption components (*p* < 0.001), while men showed greater physical activity and social participation. Women reported poorer sleep metrics (efficiency, latency, duration) and higher insomnia severity (all *p* < 0.05). Psychological distress was more prevalent among women, who also expressed greater needs for psychosocial and nutritional support (*p* < 0.001). The MedLifeMEDLIFE Index showed significant positive correlations with physical activity (*r* = 0.298), social participation (*r* = 0.227), and sleep satisfaction (*r* = 0.181), and negative correlations with mental health measures (insomnia: *r* = –0.137; depression: *r* = –0.115; stress: *r* = –0.089; anxiety: *r* = –0.076; all *p* < 0.001).

**Conclusion:**

Our findings reveal distinct gender-specific patterns in MedDiet adherence and associated lifestyle factors. These results underscore the need for differentiated public health approaches that address the unique behavioral and psychosocial needs of men and women to promote MedLife adoption.

## 1 Introduction

In recent years, scientific interest in dietary patterns impacting global health has surged, with a focus on traditional diets linked to significant reductions in cardiovascular mortality and chronic disease risk (1, 2). Among these, the Mediterranean Diet (MedDiet) is the most extensively studied and is recognized for its association with increased longevity and reduced incidence of type 2 diabetes, neurodegenerative diseases, and certain cancers (3, 4). Originating from the work of Ancel Keys in the Seven Countries Study (5), the MedDiet reflects the traditional eating habits of Mediterranean regions in the 1960s—a period marked by low incidence of diet-related diseases and high life expectancy (6, 7).

Martínez-González et al. (8) described how the MedDiet prioritizes plant-based foods such as fruits, vegetables, whole cereals, legumes, and seeds, with olive oil as the primary fat source - a pattern now shown to reduce systemic inflammation and oxidative stress (9). Red meat and refined foods are consumed in moderation, while poultry, fish, dairy, and moderate red wine consumption complete this dietary pattern, contributing to its distinctive cultural dimension and cardioprotective effects (10).

The health benefits of the MedDiet are supported by a substantial body of research, including population-based studies and clinical trials (11, 12). Notably, the PREDIMED study demonstrated a 30% reduction in major cardiovascular events among those who adhered to a MedDiet supplemented with extra-virgin olive oil or walnuts (8, 13). Systematic reviews and meta-analyses have shown the MedDiet’s impact on reducing risks of various chronic diseases, including obesity (12, 14), hypertension (15), metabolic syndrome (16), cardiovascular diseases (17), neurodegenerative disorders (18), and specific cancers (19).

The MedDiet is valued for its protective role against non-communicable diseases, attributed to its high fiber content, low glycemic index, and abundance of antioxidant and anti-inflammatory compounds (4). However, globalization, urbanization, and Western dietary influences, characterized by ultra-processed foods, refined sugars, and unhealthy fats, have led to a decline in adherence to the MedDiet, both in Mediterranean and non-Mediterranean regions (20, 21). Economic challenges, lifestyle changes, and a preference for convenience foods over traditional home-cooked meals have particularly affected younger generations (22–24).

Adherence to the MedDiet is influenced by socio-demographic factors, including socioeconomic status, education level, and employment type (25). Studies indicate that lower socioeconomic status is associated with higher consumption of refined cereals and added fats, while higher socioeconomic groups show greater consumption of fruits, vegetables and whole grains (26). Barriers such as financial constraints, limited access to nutritious foods, and disparities in dietary knowledge affect adherence, particularly in economically disadvantaged communities (27).

Despite well-documented health benefits, research on gender differences in MedDiet adherence remains inconclusive. While some studies show women have greater adherence due to higher intake of plant-based foods (28), others report no significant gender differences (29). These contradictory findings suggest complex interactions between biological, psychological and cultural factors that require further analysis.

As part of the broader MEDIET4ALL PRIMA project supported by the European Union, which aims to promote the MedDiet and its lifestyle as a sustainable and health-focused model (30), this study aimed to evaluate adherence to the MedDiet and associated lifestyle behaviors within a large, diverse survey population. Using an evidence-based approach, the study established adherence thresholds based on percentiles derived from the surveyed population while examining gender-specific patterns and exploring relationships between adherence scores and lifestyle parameters.

## 2 Materials and methods

### 2.1 Survey design and participant recruitment

An international online survey, known as the MEDIET4ALL survey, was conducted in multiple languages to examine adherence to the MedDiet and associated lifestyle behaviors. The survey was developed by a multidisciplinary team of researchers in public health, nutrition, psychology, and social sciences, who used validated instruments to ensure cultural sensitivity and reliability. Items without official translations underwent rigorous translation and back-translation processes, ensuring high test–retest reliability coefficients (*r* = 0.81–0.94) for all translated items. The survey was disseminated across ten Mediterranean and neighboring countries (Germany, France, Italy, Spain, Luxembourg, Tunisia, Algeria, Morocco, Turkey, and Jordan) during the summer of 2024 for a period of four months.

**Figure 1:**
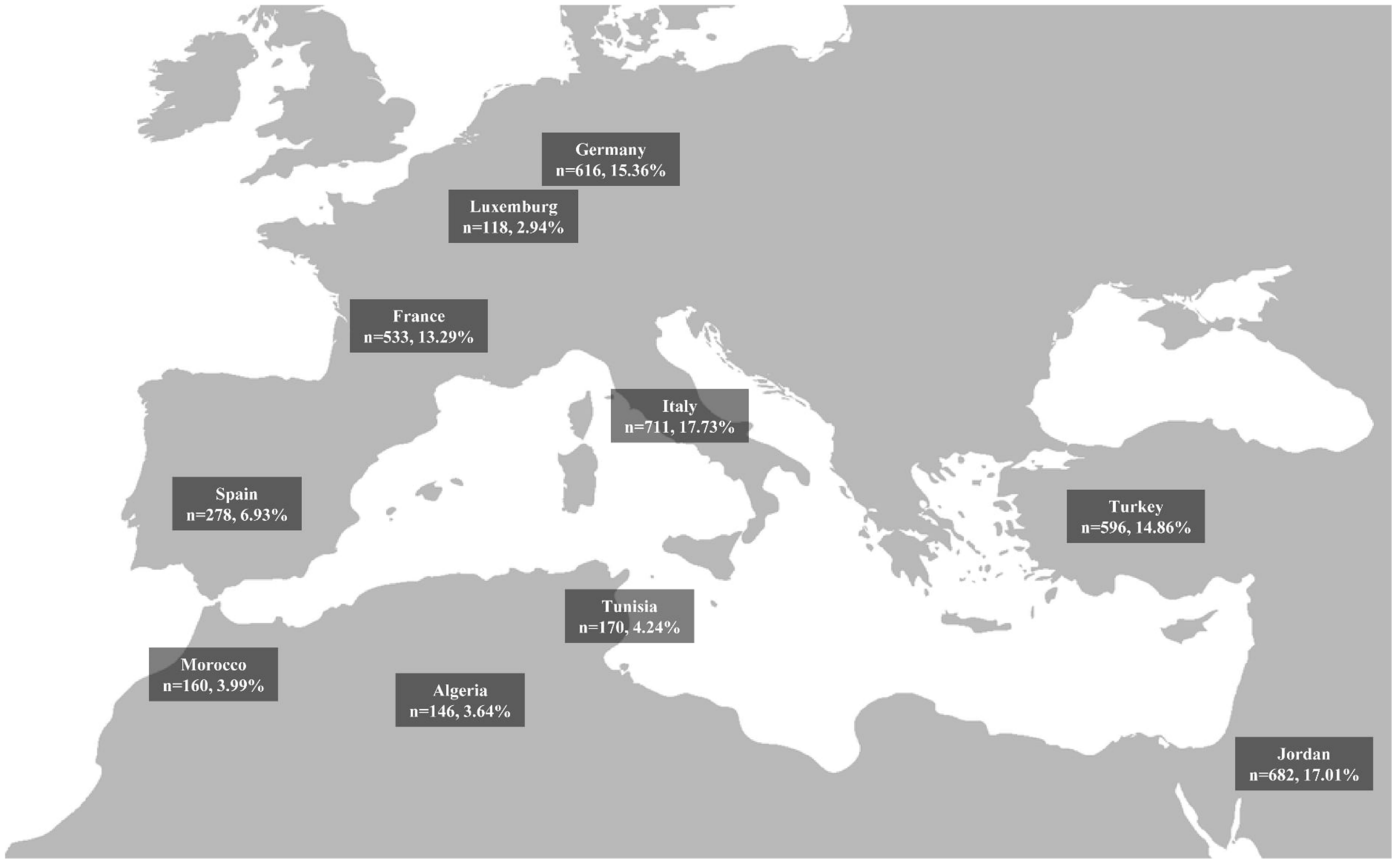
Geographical distribution and sample sizes of study participants from selected mediterranean and non-mediterranean countries.

The survey initially attracted more than 8,000 participants from various regions. After screening for validity and completeness, 4,010 responses were included in the final analysis (Figure 1). Survey completeness was evaluated by ensuring that only fully completed responses were retained, while those with missing data were excluded. To enhance data validity, responses underwent logic-based screening to detect inconsistencies or contradictions, such as claiming no engagement in vigorous physical activity (PA) while simultaneously reporting daily participation in such exercises. Additionally, duplicate entries were identified and removed using a combination of criteria, including matching IP addresses, closely timed submissions, and highly similar demographic and response patterns. Extreme or implausible values, such as reporting unrealistically long sleep durations (e.g., 24 h) or improbable dietary intake, were also filtered out to improve data accuracy and reliability. The survey targeted a broad, general population sample to ensure diversity and improve statistical power across analyses. The survey was hosted on the SoSci Survey platform, a “General Data Protection Regulation” (GDPR) compliant web application, with support from Johannes Gutenberg University.

The MEDIET4ALL survey aimed to provide a comprehensive understanding of dietary adherence and lifestyle patterns by collecting data on demographic and behavioral factors, including dietary behaviors, PA, social participation, sleep quality, mental health, and socio-demographic characteristics. Several validated questionnaires were utilized to assess adherence to the MedDiet and related lifestyle factors, including the MedLife Index (27) for dietary adherence, and others for assessing PA (IPAQ-SF), mental health (DASS-21), and life satisfaction (SLSQ).

The survey was available in seven languages: English, German, French, Italian, Spanish, Arabic, and Turkish, ensuring accessibility for participants across various regions. The survey was disseminated by the MEDIET4ALL consortium and collaborators (e.g., Bilendi solution) through email invitations, university and consortium websites, and social media platforms such as ResearchGate™, LinkedIn™, Facebook™, WhatsApp™, and Twitter™. Additionally, the general public was encouraged to promote the survey within their personal networks.

The survey began with an introductory page that outlined the study’s background, objectives, ethics, data privacy, and consent information, with participants having the option to choose one of the seven available languages. The completion of this survey was estimated to take 15–20 min.

The study was conducted in compliance with the principles outlined in the Declaration of Helsinki, and the protocol and consent form were approved by the Ethics Committee of the Faculty of Medicine at the University of Sfax (approval identification code: 066/24).

Participation was entirely voluntary, and participants were informed that all data would be used solely for research purposes and would remain anonymous and confidential in accordance with the SoSci Survey privacy policy (www.soscisurvey.de/en/privacy). The survey adhered to the Federal Data Protection Act (BDSG) and the EU General Data Protection Regulation (GDPR). Participants were not asked to provide personally identifiable information, and their responses were recorded only upon submitting the survey. Withdrawal was permitted at any stage, with no negative consequences for participants. By completing the survey, participants provided informed consent for the anonymous use of their data for research purposes. Note: While the survey recorded binary male/female categories, we use the term ‘gender’ throughout this manuscript to reflect behavioral and sociocultural dimensions aligned with the public health approach and EU gender-sensitive research policies.

### 2.2 Measures and questionnaires

Demographic data—including age, biological gender, educational level, and self-rated health status—were collected alongside a comprehensive set of validated instruments to assess adherence to the MedDiet and related lifestyle factors, as outlined by Boujelbane et al. (31). All tools were selected based on their proven reliability and validity in international populations (31).

### 2.3 MedDiet assessment

MedLife adherence was measured using the MedLife Index, a 28-item questionnaire that evaluates three key dimensions: food consumption frequency (15 items), dietary habits (7 items), and lifestyle behaviors (6 items) (32). This tool has demonstrated good internal consistency (Cronbach’s *α* = 0.75) in previous validation studies (32–34). Participants were categorized into low (<12), moderate (12–16), or high (>16) adherence groups based on tertile distributions of total scores (range 0–28) (31).

### 2.4 Sleep evaluation

Sleep characteristics were assessed using two validated measures. First, four core components from the Pittsburgh Sleep Quality Index (PSQI) were used to examine sleep efficiency (<85% vs. ≥ 85% time asleep/time in bed), latency (<20 vs. ≥ 20 min to fall asleep), subjective quality (rated on a 4-point Likert scale), and duration (7–9 h for participants under 65 years; 7–8 h for those 65 and older) (35, 36). Second, the Insomnia Severity Index (ISI) provided a comprehensive evaluation of insomnia symptoms across seven domains, including sleep onset, maintenance, early awakening, and daytime consequences, with total scores ranging from 0–28 (37).

### 2.5 Mental health assessment

Psychological wellbeing was evaluated using the Depression Anxiety Stress Scales-21 (DASS-21), which contains three 7-item subscales that measure symptoms of depression, anxiety, and stress experienced during the previous week using a 4-point Likert scale (38).

### 2.6 Psychosocial and behavioral measures

Life satisfaction was assessed with the Short Life Satisfaction Questionnaire-Lockdown (SLSQ), a 3-item adaptation of the Satisfaction with Life Scale that uses a 7-point Likert scale (39, 40). PA patterns were quantified using the International Physical Activity Questionnaire Short Form (IPAQ-SF), which calculates weekly energy expenditure in MET-minutes across vigorous, moderate, and walking activity domains (41, 42). Social interaction patterns were evaluated with the 14-item Short Social Participation Questionnaire-Lockdowns (SSPQL), while technology usage was measured using the 3-item Short Technology-Use Questionnaire-Lockdowns (STuQL) (31, 39, 40).

### 2.7 Barriers to adherence

The study incorporated the MedDiet Barriers Questionnaire (MBQ), a novel 13-item tool that systematically evaluates potential adherence barriers across four domains: physiological, socioeconomic, cultural-attitudinal, and practical constraints using a dichotomous (yes/no) response format (31).

### 2.8 Statistical analysis

The statistical analyses were performed using SPSS version 25. The percentage presented in the tables represents the responses for males and females in each item, calculated relative to the total male and female responses. Descriptive statistics were calculated to determine the distribution of MedLife adherence categories across geo-demographic and socio-economic factors. A chi-square test of independence (χ^2^) was conducted to examine the relationships between gender and various categorical variables. To assess whether the distribution of participants across categorical variables differed significantly by gender, a Z-test for two proportions was conducted using the pooled variance formula, as applied in previous research [e.g., Boujelbane et al. (31)]. This test accounts for the imbalanced sample sizes between genders, with significance thresholds set at ∣Z∣ ≥ 1.96 (*p* < 0.05), ∣Z∣ ≥ 2.58 (*p* < 0.01), and ∣Z∣ ≥ 3.29 (*p* < 0.001) (31).

The Shapiro–Wilk test was applied to assess data normality, revealing that the data were not normally distributed. Therefore, the Mann–Whitney test was used to compare total scores between males and females. Spearman’s rank-order correlation was employed to assess the relationship between the MedLife index and other scores. The significance level for all tests was set at 5% (*p* < 0.05).

## 3 Results

This study included 4,010 participants from 10 countries, with a mean age of 37.24 ± 15.38 years overall. Females accounted for 59.5% of the sample, with a mean age of 36.04 ± 15.06 years. Males had a mean age of 39.02 ± 15.68 years.

### 3.1 Demographic characteristics of the participants

The demographic characteristics of the participants are presented in Table 1. The majority resided in urban areas, with no statistically significant gender-based difference in residential distribution (*p* = 0.756). However, a pronounced gender disparity was observed in continental representation (*p* < 0.001). Participants of both genders were predominantly from Europe (*p* < 0.001), with a higher proportion among males (*p* < 0.001) compared to females. Females had higher presentiveness in Asia (*p* < 0.001) and Africa (*p* < 0.01). Ethnic background also showed significant gender differences (*p* < 0.001). The most represented ethnic group was White/European, with a significantly higher proportion among males compared to females. This was followed by Middle Eastern/Arab participants, where females were more represented, and Turks participants, with a significantly higher proportion among males (*p* < 0.001).

**Table 1:** Sociodemographic characteristics of the study population (*n* = 4,010) stratified by gender.

Variables	Male	Female	Total	*χ* ^2^	df	*p*
Country of living						
Algeria	58 (3.6%)	88 (3.7%)	146	266.41	9	<0.0001
France	244 (15%)	289 (12.1%)**	533			
Germany	290 (17.8%)	326 (13.7%)***	616			
Italy	351 (21.6%)	360 (15.1%)***	711			
Luxembourg	43 (2.6%)	75 (3.1%)	118			
Tunisia	84 (5.2%)	86 (3.6%)*	170			
Spain	96 (5.9%)	182 (7.6%)*	278			
Morocco	79 (4.9%)	81 (3.4%)*	160			
Turkey	284 (17.5%)	312 (13.1%)***	596			
Jordan	96 (5.9%)	586 (24.6%)***	682			
Region						
MC	429 (26.4%)	987 (41.4%)***	1,416	94.99	1	<0.0001
NMC	1,196 (73.6%)	1,398 (58.6%)***	2,594			
Continent						
Europe	1,024 (63%)	1,232 (51.7%)***	2,256	90.78	2	<0.0001
Asia	380 (23.4%)	898 (37.7%)***	1,278			
Africa	221 (13.6%)	255 (10.7%)**	476			
Ethnicity						
Prefer not to say	90 (5.5%)	111 (4.7%)	201	150.41	7	<0.0001
Black/African/Caribbean	54 (3.3%)	71 (3%)	125			
Latin American/Hispanic	28 (1.7%)	34 (1.4%)	62			
White/ European	879 (54.1%)	1,061 (44.5%)***	1940			
Asian	40 (2.5%)	70 (2.9%)	110			
Middle Eastern/Arab	212 (13%)	694 (29.1%)***	906			
Turks	272 (16.7%)	278 (11.7%)***	550			
Other	50 (3.1%)	66 (2.8%)	116			
Living environment						
Urban environment	1,072 (66%)	1,586 (66.5%)	2,658	0.558	2	0.756
Suburban environment	291 (17.9%)	435 (18.2%)	726			
Rural environment	262 (16.1%)	364 (15.3%)	626			
Age (years)						
18–35	818 (50.3%)	1,352 (56.7%)***	2,170	23.11	2	<0.0001
36–55	486 (29.9%)	685 (28.7%)	1,171			
> 55	321 (19.8%)	348 (14.6%)***	669			
BMI						
Underweight:	50 (3.1%)	159 (6.7%)***	209	91.97	3	<0.0001
Normal weight	767 (47.2%)	1,379 (57.8%)***	2,146			
Overweight:	790 (48.6%)	824 (34.5%)***	1,614			
Obesity	18 (1.1%)	23 (1%)	41			
Education						
No schooling completed	96 (5.9%)	112 (4.7%)	208	38.17	4	<0.0001
High school graduate, diploma or the equivalant	457 (28.1%)	571 (23.9%)**	1,028			
Professional degree	181 (11.1%)	235 (9.9%)	416			
Bachelor’s degree	498 (30.6%)	957 (40.1%)***	1,455			
Master- doctorte degree	393 (24.2%)	510 (21.4%)*	903			
Marital status						
Single	790 (48.6%)	1,183 (49.6%)	1973	7.22	2	0.027
Married living as couple	736 (45.3%)	1,010 (42.3%)	1746			
Widowed, divorced, separated	99 (6.1%)	192 (8.1%)*	291			
Employment						
Employed	924 (56.9%)	1,109 (46.5%)***	2033	118.77	4	<0.0001
Unmployed	95 (5.8%)	372 (15.6%)***	467			
Student	381 (23.4%)	646 (27.1%)**	1,027			
Retired	171 (10.5%)	168 (7%)***	339			
Uncategorized	54 (3.3%)	90 (3.8%)	144			
Health status						
Healthy	1,221 (75.1%)	1793 (75.2%)	3,014	3.87	2	0.144
At risk	301 (18.5%)	407 (17.1%)	708			
With diseases	103 (6.3%)	185 (7.8%)	288			
Smoking						
Cigarettes smokers	427 (26.3%)	368 (15.4%)***	795	73.62	2	<0.0001
Shisha smokers	68 (4.2%)	141 (5.9%)*	209			
Non-smokers	1,130 (69.5%)	1876 (78.7%)***	3,006			

Significantly different from males: *p < 0.05; **p < 0.01; ***p < 0.001.

Age distribution revealed notable gender differences (*p* < 0.001), with the majority of respondents being young adults aged 18–35 years. Females were predominantly within this age range, while males showed higher representation among older age groups (*p* < 0.001).

BMI classification also indicated significant gender differences (*p* < 0.001). Normal weight was the most common category, particularly among females (*p* < 0.001). Furthermore, females were more frequently classified as underweight, whereas males had a higher prevalence of overweight (*p* < 0.001). Obesity rates were low in both sexes.

Educational attainment varied significantly by gender (*p* < 0.001). The most common categories were high school graduates, bachelor’s degree holders, and individuals with a master’s or doctoral degree. Females were more likely to have completed a bachelor’s degree (*p* < 0.001), while males were more frequently high school graduates (*p* < 0.01) and more represented among those with a master’s degree (*p* < 0.05). A slightly higher proportion of males also reported participation in professional training programs.

Marital status distribution differed significantly between sexes (*p* = 0.027), with a higher number of females reporting being widowed, divorced, or separated (*p* < 0.05).

Employment status also showed significant gender differences (*p* < 0.001). While most participants were employed, males were more likely to be employed (*p* < 0.001). Females were more frequently unemployed (*p* < 0.001) or students (*p* < 0.01), whereas males had higher representation in the retired category (*p* < 0.001).

Self-reported health status did not differ significantly by gender (*p* = 0.144). The majority reported being in good health, and the proportions of participants “at risk of developing a disease” or “with existing diseases” were similar across gender.

Smoking behavior showed significant gender disparities (*p* < 0.001). Males had a higher prevalence of cigarette smoking (*p* < 0.001), whereas females showed a greater prevalence of shisha smoking (*p* < 0.05). Overall, non-smoking was the most common behavior, with a significantly higher proportion of females identifying as non-smokers (*p* < 0.001).

### 3.2 Behavioral characteristics of the participants

#### 3.2.1 MedLife index and perceived barriers

**Figure 2:**
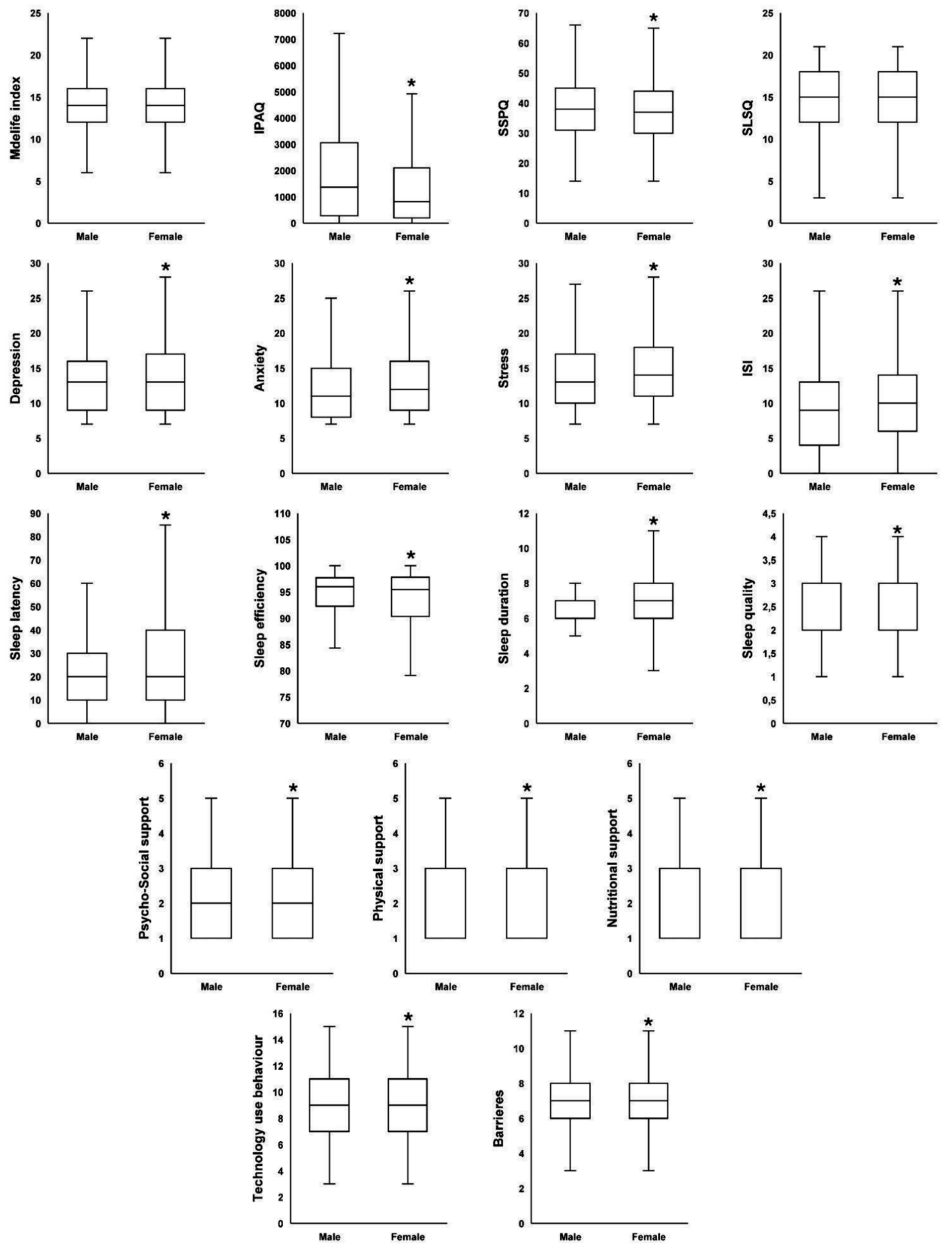
Gender differences in total scores for health and lifestyle parameters. SLSQ-L, short life satisfaction questionnaire; ISI, insomnia severity index; IPAQ, the international physical activity questionnaires; SSPQ-L, short social participation questionnaire; *significantly different compared to males at *p* < 0.05.

The Mann–Whitney analysis of the total MedLife Index score (Figure 2) revealed no significant gender differences (*Z* = −1.2, *p* = 0.219, Cohen’s *d* = 0.04). A more detailed analysis (Table 2) showed that in Block 1 (Mediterranean Food Consumption), females scored significantly higher than males (*Z* = −4.83, *p* < 0.001, Cohen’s *d* = 0.16). This difference was driven by significant differences in most parameters: out of the 15 total items, females adhered more closely to the recommended intake in 7 items (red meat, processed meat, Eggs, vegetables, olive oil, herbs/spices/garnish, potatoes; *p* < 0.001), while males showed higher adherence in 3 items (fish/seafood, sweets, Legumes; *p* = 0.001). No significant gender differences were observed in the remaining 5 items (white meat, low-fat dairy products, nuts and olives, fruit, cereals; *p* > 0.05).

**Table 2:** Analysis of the MedLife Index: Mediterranean diet adherence, dietary habits, and consumption patterns by gender.

MedLife index		*n*			Gender effect		
Food Group	Criteria for 1 point	Male	Female	Total			
Block 1: Mediterranean food consumption					*χ* ^2^	df	*p*
Sweets	≤2 servings/week	969 (59.6%)	1,292 (54.2%)***	2,261	11.71	1	0.001
Red meat	<2 servings/week	716 (44.1%)	1,271 (53.3%)***	1987	32.94	1	<0.0001
Processed meat	≤1 serving/week	812 (50%)	1,459 (61.2%)***	2,271	49.41	1	<0.0001
Eggs	2–4 servings/week	660 (40.6%)	1,166 (48.9%)***	1862	26.68	1	<0.0001
Legumes	≥2 servings/week	1,035 (63.7%)	1,395 (58.5%)***	2,430	10.95	1	0.001
White meat	2 servings/week	387 (23.8%)	547 (22.9%)	934	0.42	1	0.517
Fish/seafood	≥2 servings/week	602 (37%)	700 (29.4%)***	1,302	26.11	1	<0.0001
Potatoes	≤3 servings/week	1,320 (81.2%)	2037 (85.4%)***	3,357	12.38	1	<0.0001
Low-fat dairy products	2 servings/d	253 (15.6%)	329 (13.8%)	582	2.45	1	0.117
Nuts and olives	1–2 servings/d	893 (55%)	1,292 (54.2%)	2,185	0.238	1	0.625
Herbs, spices and garnish	≥1 serving/d	1,442 (88.7%)	2,204 (92.4%)***	3,646	15.79	1	<0.0001
Fruit	3–6 servings/d	287 (17.7%)	406 (17%)	693	0.28	1	0.600
Vegetables	≥2 servings/d	744 (45.8%)	1,252 (52.5%)***	1996	17.41	1	<0.0001
Olive oil	≥3 servings/d	403 (24.8%)	816 (34.2%)***	1,219	40.48	1	<0.0001
Cereals	3–6 servings/d	392 (24.1%)	589 (24.7%)	981	0.17	1	0.679

Block 1: Total score	Male	Female		*Z*	*p*	Cohen’s *d*
	6.72 ± 1.98	7.03 ± 1.91		−4.83	0.000	0.16

Block 2: Mediterranean dietary habits					*χ* ^2^	df	*p*
Water or infusions	6–8 servings/d or ≥3 servings/week	1,348 (83%)	1819 (76.3%)***	3,167	26.02	1	<0.0001
Wine	1–2 servings/d	215 (13.2%)	135 (5.7%)***	350	69.53	1	<0.0001
Limit salt in meals	Yes	1,060 (65.2%)	1,483 (62.2%)*	2,543	3.88	1	0.049
Preference for whole grain products	Yes/fibre > 25 g/d	917 (56.4%)	1,473 (61.8%)***	2,390	11.4	1	0.001
Snacks	≤2 servings/week	1,154 (71%)	1717 (72%)	2,871	0.453	1	0.501
Limit nibbling between meals	Yes	1,006 (61.9%)	1,551 (65%)*	2,557	4.08	1	0.043
Limit sugar in beverages (including sugar-sweetened beverages)	Yes	1,162 (71.5%)	1793 (75.2%)**	2,955	6.72	1	0.010
Processed food	Yes	1,298 (79.9%)	2000 (83.9%)**	3,298	10.49	1	0.001
Moderation trying	Yes	917 (56.4%)	1,639 (68.7%)***	2,556	63.17	1	<0.0001

Block 2: Total score	Male	Female	*Z*	*p*	Cohen’s *d*
	4.22 ± 1.56	4.18 ± 1.55	−0.62	0.538	0.03

Block 3: Physical activity, rest, social habits and conviviality					*χ* ^2^	df	*p*
Physical activity (>150 min/week or 30 min/d)	Yes	1,079 (66.4%)	1,340 (56.2%)***	2,419	42.14	1	<0.0001
Siesta/nap	Yes	666 (41%)	917 (38.4%)	1,583	2.6	1	0.107
Hours of sleep	6–8 h/d	1,176 (72.4%)	1,665 (69.8%)	2,841	3.06	1	0.080
Watching television	<1 h/d	301 (18.5%)	519 (21.8%)*	820	6.23	1	0.013
Socializing with friends	≥2 h/weekend	1,257 (77.4%)	1,635 (68.6%)***	2,892	37.23	1	<0.0001
Collective sports	≥2 h/week	631 (38.8%)	500 (21%)***	1,131	152.36	1	<0.0001
Company	Yes	1,192 (73.4%)	1918 (80.4%)***	3,110	27.72	1	<0.0001

Block 3: Total score	Male	Female	*Z*	*p*	Cohen’s *d*
	3.15 ± 1.27	2.76 ± 1.24	−9.3	0.000	0.31

MedLife categories	Male	Female	Total	*χ* ^2^	df	*p*
Low	495 (30.5%)	753 (31.6%)	1,248 (31.1%)	1.57	2	0.457
Medium	755 (46.5%)	1,120 (47%)	1875 (46.8%)			
High	375 (23.1%)	512 (21.5%)	887 (22.1%)			

MedLife index total score		Male	Female	*Z*	*p*	Cohen’s *d*
		14.08 ± 3.21	13.96 ± 3.11	−1.2	0.219	0.04

Significantly different from males: *p < 0.05; **p < 0.01; ***p < 0.001.

In Block 2 (Mediterranean Dietary Habits), no significant gender differences were observed in the overall score (*Z* = −0.62, *p* = 0.538, Cohen’s *d* = 0.03). However, a more detailed item-level analysis using the *Z*-test for two proportions (Table 2) revealed significant gender-specific adherence patterns. Females showed greater adherence to recommendations related to whole grain consumption (*p* < 0.001), limiting snacking between meals (*p* < 0.05), and reducing sugar intake in beverages (*p* < 0.01). In contrast, males demonstrated better adherence to recommendations concerning water or infusion consumption, wine intake (*p* < 0.001), and salt limitation (*p* < 0.05).

In Block 3 (PA, Rest, Social Habits, and Conviviality), males scored significantly higher than females (*Z* = −9.3, *p* < 0.001, Cohen’s *d* = 0.31). Out of the 6 total items, males followed the recommended activity more frequently in 3 items (PA, collective sports, socializing with friends; *p* < 0.001), whereas females showed higher adherence in one item (watching television; *p* < 0.01). No significant gender differences were found in 2 items (siesta/nap, hours of sleep; *p* > 0.05).

Regarding MedLife Index categories (low, medium, and high adherence; Table 2), gender had no significant impact [χ^2^(2, N = 4,010) = 1.57, *p* > 0.05]. Post-hoc analysis confirmed that there were no significant differences in the proportion of males and females across the three MedLife Index categories.

Regarding the impact of gender on perceived barriers, the Mann–Whitney test revealed that females reported significantly higher perceived barriers compared to males (*Z* = 2.08, *p* = 0.037, Cohen’s *d* = 0.08) (Figure 2). A more detailed analysis of gender differences for each item (Table 3) showed significant differences in five specific barriers: a higher proportion of females reported attitude-related barriers (*p* < 0.001), lack of knowledge (*p* < 0.05), and taste dislike (*p* < 0.001), while males reported higher proportions in low motivation (*p* < 0.05) and medical reasons (*p* < 0.001).

**Table 3:** Gender differences in awareness and potential barriers influencing their adherence to MedLife.

	Yes: *n* (%)			gender effect		
Variables	Male	Female	Total	*χ* ^2^	df	*p*
Awarness	894 (55%)	1,291 (54.1%)	2,185	0.31	1	0.580
Attitudes	1,287 (79.2%)	1995 (83.6%)***	3,282	12.87	1	<0.0001
Social norms	1,333 (82%)	1999 (83.8%)	3,332	2.19	1	0.139
Low motivation	1,392 (85.7%)	1982 (83.1%)*	3,311	1.17	1	0.280
Price affordability	1,184 (72.9%)	1,693 (71%)	2,877	1.68	1	0.195
Time/ effort consuming	1,224 (75.3%)	1812 (76%)	3,036	0.22	1	0.637
Low accessibility	1,393 (85.7%)	2066 (86.6%)	3,459	0.663	1	0.416
Lack of knowledge	1,309 (80.6%)	1979 (83%)*	3,288	3.84	1	0.050
Food allergies and intolerances	207 (12.7%)	333 (14%)	540	1.24	1	0.265
Cultural and/or religious reason	172 (10.6%)	249 (10.4%)	421	0.02	1	0.884
Medical reason	203 (12.5%)	212 (8.9%)***	415	13.53	1	<0.0001
Individual beliefs (e.g., vegan, vegetarian)	148 (9.1%)	215 (9%)	363	0.01	1	0.920
Taste dislike	260 (16%)	481 (20.2%)***	741	11.14	1	0.001
Others	47 (2.9%)	86 (3.6%)	133	3.83	1	0.050

Total score				Male	Female	*Z*	*p*	Cohen’s *d*
	6.81 ± 1.29	6.91 ± 1.28	2.08	0.037	0.08

Significantly different from males: *p < 0.05; **p < 0.01; ***p < 0.001.

#### 3.2.2 Physical and social activities, sleep patterns and use of technology behaviors

The Mann–Whitney test revealed that males had significantly higher IPAQ-SF scores than females (*Z* = −7.93, *p* < 0.001, Cohen’s *d* = 0.28), and scored higher on the SSPQ (*Z* = −2.38, *p* = 0.017, Cohen’s *d* = 3.43) indicating greater PA and social participation among this population. Regarding sleep patterns, males exhibited higher sleep efficiency (*Z* = −4.61, *p* < 0.001, Cohen’s *d* = 0.14), whereas females showed longer sleep latency (*Z* = −5.13, *p* < 0.001, Cohen’s *d* = 0.21), poorer sleep quality (*Z* = −2.09, *p* = 0.037, Cohen’s *d* = 0.08), longer sleep duration (*Z* = −2.18, *p* = 0.029, Cohen’s *d* = 0.06), and higher ISI scores (*Z* = −5.76, *p* < 0.001, Cohen’s *d* = 0.2). Females also reported higher overall technology use behavior (*Z* = −2.8, *p* = 0.005, Cohen’s *d* = 0.29) (Figure 2).

In terms of prevalences across categories, gender had no significant impact on social participation categories (Never to all times socially active), sleep duration categories (below, within, or exceeded recommendation), or sleep quality (very good to very bad) (*p* > 0.05) (Table 4). However, gender significantly influenced PA, sleep latency, sleep efficiency, and ISI categories (*p* < 0.001). Males were more physically active, as indicated by their lower proportion in the Lowly Active category and higher proportion in the Highly Active category compared to females (*p* < 0.001, Table 4). Additionally, males also had a lower proportion in the high (>20) sleep latency (*p* < 0.001), low (<85) sleep efficiency (*p* < 0.01), and moderate (*p* < 0.001) to severe (*p* < 0.01) insomnia categories compared to females (Table 4).

**Table 4:** Behavioural and sleep outcomes by gender.

Variables	*n*			Gender effect		
IPAQ categories	Male	Female	Total	*χ* ^2^	df	*p*
Low activities	863 (53.1%)	1,555 (65.2%)***	2,418 (60.3 %)	76.3	2	<0.0001
Moderate activities	347 (21.4%)	463 (19.4%)	810 (20.2 %)			
High activities	415 (25.5%)	367 (15.4%)***	782 (19.5 %)			

IPAQ categories				Male	Female		*Z*	*p*	Cohen’s *d*
	2130.59 ± 2445.65	1521.32 ± 1935.03		−7.93	0.000	0.28

SPQ categories	Male	Female	Total	*χ* ^2^	df	*p*
Never	9 (0.6%)	11 (0.5%)	20 (0.5 %)	7.19	4	0.126
Rarely	263 (16.2%)	446 (18.7%)*	709 (17.7 %)			
Sometimes	824 (50.7%)	1,211 (50.8%)	2,035 (50.8%)			
Often	451 (27.8%)	629 (26.4%)	1,080 (26.9 %)			
All times	78 (4.8%)	88 (3.7%)	166 (4.1 %)			

SPQ total score				Male	Female	*Z*	*p*	Cohen’s *d*
	38.36 ± 10.21	37.6 ± 9.96	−2.38	0.017	30.43

Sleep						
Efficiency	Male	Female	Total	*χ* ^2^	df	*p*
*> 85*	1,472 (90.6%)	2096 (87.9%)**	3,568	7.2	1	0.007
*< 85*	153 (9.4%)	289 (12.1%)**	442			

Sleep efficiency				Male	Female	*Z*	*p*	Cohen’s *d*
	93.73 ± 6.78	92.76 ± 7.38	−4.61	0.000	0.14

Latency	Male	Female	Total	*χ* ^2^	Df	*p*
<20	1,011 (62.2%)	1,322 (55.4%)***	2,333	18.29	1	<0.0001
>20	614 (37.8%)	1,063 (44.6%)***	1,677			

Sleep latency				Male	Female	*Z*	*p*	Cohen’s *d*
	27.57 ± 31.76	32.33 ± 34.81	−5.13	0.000	0.21

Quality	Male	Female	Total	*χ* ^2^	df	*p*
Very good	308 (19%)	407 (17.1%)	715	5.34	3	0.149
Fairly good	844 (51.9%)	1,226 (51.4%)	2070			
Fairly bad	395 (24.3%)	607 (25.5%)	1,002			
Very bad	78 (4.8%)	145 (6.1%)	223			

Sleep quality				Male	Female	*Z*	*p*	Cohen’s *d*
	2.15 ± 0.78	2.21 ± 0.79	−2.09	0.037	0.08

Duration	Male	Female	Total	*χ* ^2^	df	*p*
Below recommendation	724 (44.6%)	1,006 (42.2%)	1730	3.99	2	0.135
Within recommendation	815 (50.2%)	1,223 (51.3%)	2038			
Exceeded recommendation	86 (5.3%)	156 (6.5%)	242			

Sleep duration				Male	Female	*Z*	*p*	Cohen’s *d*
	6.76 ± 1.66	6.86 ± 1.73	−2.18	0.029	0.06

Insomnia	Male	Female	Total	*χ* ^2^	*df*	*p*
Absence of insomnia	676 (41.6%)	862 (36.1%)***	1,538	28.07	3	<0.0001
Sub-threshold insomnia	662 (40.7%)	954 (40%)	1,616			
Moderate insomnia	257 (15.8%)	484 (20.3%)***	741			
Severe insomnia	30 (1.8%)	85 (3.6%)**	115			

ISI: Total score				Male	Female	*Z*	*p*	Cohen’s *d*
	8.99 ± 5.68	10.13 ± 5.88	−5.76	0.000	0.2

Technology use behavior	Male	Female	Total	*χ* ^2^	*df*	*p*
Rarely	82 (5%)	92 (3.9%)	174	7.9	3	0.048
Sometimes	254 (15.6%)	340 (14.3%)	594			
Often	670 (41.2%)	957 (40.1%)	1,627			
All times	619 (38.1%)	996 (41.8%)*	1,615			

Technology use behavior				Male	Female	*Z*	*p*	Cohen’s *d*
	8.74 ± 2.76	9 ± 2.72	−2.8	0.005	0.29

IPAQ-SF, the international physical activity questionnaires-short form; SSPQ-L, short social participation questionnaire; ISI, insomnia severity index. Significantly different from males: *p < 0.05; **p < 0.01; ***p < 0.001.

#### 3.2.3 Life satisfaction, mental health (DASS-21), and the need for psychosocial, physical, and nutritional support

Gender did not have a significant impact on overall life satisfaction scores (*p* > 0.05). However, significant gender differences were observed in depression, anxiety, and stress levels, as well as in the need for psycho-social, physical, and nutritional supports (*p* < 0.001). (Table 5).

**Table 5:** Assessment of life satisfaction, mental health (DASS-21), and the demand for psychosocial, physical, and nutritional support.

Variables	*n*			Gender effect		
Life satisfaction questionnaire	Male	Female	Total	*χ* ^2^	df	*p*
Extremely dissatisfied	40 (2.5%)	59 (2.5%)	99	4.53	6	0.605
Dissatisfied	64 (3.9%)	103 (4.3%)	167			
Slightly dissatisfied	137 (8.4%)	235 (9.9%)	372			
Neutral	395 (24.3%)	591 (24.8%)	986			
Slightly satisfied	357 (22%)	528 (22.1%)	885			
Satisfied	342 (21%)	485 (20.3%)	827			
Extremely satisfied	290 (17.8%)	384 (16.1%)	674			

Life satisfaction questionnaire score				Male	Female	*Z*	*p*	Cohen’s *d*
	14.34 ± 4.52	14.21 ± 4.51	−1.05	0.293	0.03

DASS 21						
Depression	Male	Female	Total	*χ* ^2^	df	*p*
Normal	504 (31%)	599 (25.1%)***	1,103	20.12	4	<0.0001
Mild	416 (25.6%)	703 (29.5%)**	1,119			
Moderate	558 (34.3%)	829 (34.8%)	1,387			
Severe	137 (8.4%)	234 (9.8%)	371			
Extremely severe	10 (0.6%)	20 (0.8%)	30			

Depression score				Male	Female	*Z*	*p*	Cohen’s *d*
	13.06 ± 4.98	13.57 ± 5.05	−3.26	0.001	0.11

Anxiety	Male	Female	Total	*χ* ^2^	df	*p*
Normal	324 (19.9%)	317 (13.3%)***	641	36.7	4	<0.0001
Mild	347 (21.4%)	492 (20.6%)	839			
Moderate	532 (32.7%)	858 (36%)*	1,390			
Severe	287 (17.7%)	467 (19.6%)	754			
Extremely severe	135 (8.3%)	251 (10.5%)*	386			

Anxiety score				Male	Female	*Z*	*p*	Cohen’s *d*
	11.95 ± 4.7	12.59 ± 4.74	−4.94	0.000	0.19

Stress	Male	Female	Total	*χ* ^2^	df	*p*
Normal	1,014 (62.4%)	1,312 (55%)***	2,326	26.87	3	<0.0001
Mild	345 (21.2%)	580 (24.3%)*	934			
Moderate	240 (14.8%)	441 (18.5%)**	681			
Severe	17 (1%)	52 (2.2%)**	69			
Extremely severe	0 (0%)	0	0			

Stress score				Male	Female	*Z*	*p*	Cohen’s *d*
	13.57 ± 4.7	14.57 ± 4.79	−6.37	0.000	0.29

Need of support						
Psycho-social support	Male	Female	Total	*χ* ^2^	df	*p*
Never	636 (39.1%)	557 (23.4%)***	1,193	141.6	4	<0.0001
Rarely	414 (25.5%)	583 (24.4%)	997			
Sometimes	398 (24.5%)	860 (36.1%)***	1,258			
Often	129 (7.9%)	278 (11.7%)***	407			
All times	48 (3%)	107 (4.5%)*	155			

Psycho-social support score				Male	Female	*Z*	*p*	Cohen’s *d*
	2.1 ± 1.1	2.5 ± 1.1	−11.45	0.000	0.36

Physical support	Male	Female	Total	*χ* ^2^	df	*p*
Never	605 (37.2%)	524 (22%)***	1,129	134.71	4	<0.0001
Rarely	322 (19.8%)	437 (18.3%)	759			
Sometimes	423 (26%)	812 (34%)***	1,235			
Often	197 (12.1%)	431 (18.1%)***	628			
All times	78 (4.8%)	181 (7.6%)***	259			

Physical support score				Male	Female	*Z*	*p*	Cohen’s *d*
	2.28 ± 1.22	2.71 ± 1.21	−11.23	0.000	0.35

Nutritional support	Male	Female	Total	*χ* ^2^	df	*p*
Never	536 (33%)	545 (22.9%)***	1,081	86.94	4	<0.0001
Rarely	355 (21.8%)	454 (19%)*	809			
Sometimes	453 (27.9%)	727 (30.5%)	1,180			
Often	184 (11.3%)	427 (17.9%)***	611			
All times	97 (6%)	232 (9.7%)***	329			

Nutritional support score				Male	Female	*Z*	*p*	Cohen’s *d*
	2.36 ± 1.21	2.73 ± 1.26	−9.26	0.000	0.3

Significantly different from males: *p < 0.05; **p < 0.01; ***p < 0.001.

Specifically, females reported significantly higher total scores for depression (*Z* = −3.26, *p* = 0.001, Cohen’s *d* = 0.11), anxiety (*Z* = −4.94, *p* < 0.001, Cohen’s *d* = 0.19), and stress (*Z* = −6.37, *p* < 0.001, Cohen’s *d* = 0.29), indicating greater psychological distress compared to males (Figure 2). Correspondingly, males had a significantly higher proportion in the normal category for depression, anxiety, and stress (*p* < 0.001, Table 5). Furthermore, males had a significantly lower proportion in the mild depression (*p* < 0.01), mild and extremely severe anxiety (*p* < 0.05), and mild (*p* < 0.05) to severe (*p* < 0.01) stress categories (Table 5).

Regarding the need for support, females reported significantly higher scores in psycho-social support (*Z* = −11.45, *p* < 0.001, Cohen’s *d* = 0.36), physical support (*Z* = −11.23, *p* < 0.001, Cohen’s *d* = 0.35), and nutritional support (*Z* = −9.26, *p* < 0.001, Cohen’s *d* = 0.3). Males, on the other hand, were significantly more likely to report no need for psycho-social support and were less likely than females to report needing such support sometimes or often (*p* < 0.001, Table 5). A similar pattern was observed for physical support, where males were significantly more likely to report no need for support and were less likely than females to report needing support sometimes, often, or at all times (*p* < 0.001, Table 5). For nutritional support, males were again significantly more likely to report no need for support and were less likely than females to report needing support often or at all times (*p* < 0.001, Table 5).

### 3.3 Correlations between MedLife index and all other parameters

**Figure 3:**
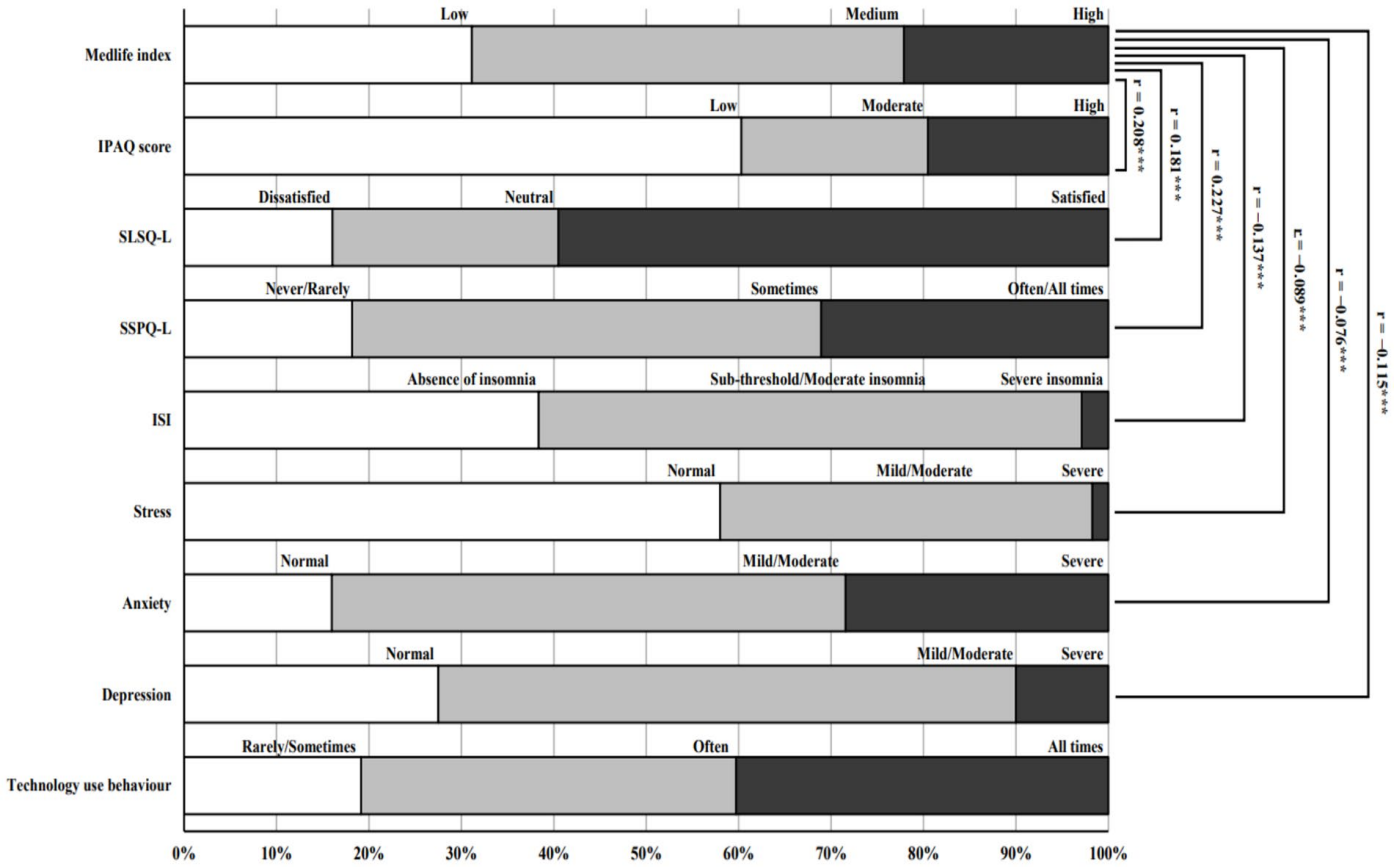
Correlations between the MedLife Index and other health and lifestyle parameters. SLSQ-L, short life satisfaction questionnaire; ISI: Insomnia Severity Index; IPAQ, the international physical activity questionnaires; SSPQ-L, short social participation questionnaire; ***significantly different compared to males at *p* < 0.001.

The analysis revealed significant correlations between the MedLife Index and various parameters (*p* < 0.001). Specifically, the MedLife Index was positively correlated with the IPAQ-SF score (*r* = 0.298), indicating that higher levels of PA are associated with better lifestyle quality. Additionally, significant positive correlations were observed with sleep satisfaction (*r* = 0.181) and perceived social support (*r* = −0.227). Conversely, the MedLife Index demonstrated significant negative correlations with insomnia severity (*r* = −0.137), stress levels (*r* = −0.089), anxiety (*r* = −0.076), and depression (*r* = −0.115). These results are presented in (Figure 3).

## 4 Discussion

Our multinational study of 4,010 participants across 10 countries provides both confirmatory evidence and novel insights through its comprehensive assessment of lifestyle interactions. In the present study, adherence to the MedDiet and its association with lifestyle behaviors were investigated within a large, diverse survey population. By defining adherence thresholds based on percentiles, the study provided a detailed analysis of dietary patterns and gender differences. Significant associations were identified between MedLife index and key lifestyle parameters, underscoring the relationship between dietary habits and lifestyle behaviors.

### 4.1 Demographic characteristics of the participants

This study reveals demographic differences that indicate underlying physiological, psychological, and sociocultural mechanisms influencing health-related behaviors. Educational attainment varied significantly by gender. Females were more likely to hold a bachelor’s degree, which may reflect their established cognitive resilience and adaptive strategies within academic settings, as evidenced by research indicating enhanced verbal and social cognitive skills that facilitate learning and collaboration (43). Although females were more represented at the bachelor’s level, males showed higher representation at the master’s level, possibly due to greater participation in male-dominated fields requiring advanced degrees (e.g., STEM, business) (44, 45), delayed entry into graduate education after work experience (46), and structural barriers such as caregiving responsibilities that may limit women’s progression to higher academic levels (47, 48). Furthermore, the slightly higher participation of males in vocational training—though not statistically significant—might stem from evolutionary predispositions favoring risk-taking and manual or technical skills, consistent with traditional gender roles (49).

Significant gender disparities were also observed in BMI classifications, with females showing a higher prevalence in the underweight and normal weight categories compared to males, while males exhibited a higher prevalence in the overweight category compared to females. This reflects fundamental biological differences in fat metabolism influenced by gender hormones, combined with social factors: women face stronger societal pressures for thinness, while men experience greater acceptance of higher body weights. Women’s generally healthier eating patterns as shown in our MedDiet results further contribute to this weight distribution pattern (50).

Gender differences in smoking behaviors, particularly the higher prevalence of non-smokers among females, may reflect physiological sensitivities to nicotine and its metabolism. Evidence suggests that males experience greater dopaminergic reinforcement from smoking, which contributes to stronger addictive patterns (51). In contrast, females may be more inclined toward alternative forms such as shisha, influenced by social context and a perception of reduced harm (51). The present findings support these observations, showing a higher prevalence of cigarette smoking among males compared to females, whereas shisha smoking was more common among females.

The increased participation of young adult females in this study may be due to their heightened engagement with health-related topics and greater responsiveness to surveys. This observation is consistent with existing research indicating that women, especially younger adults, tend to be more proactive in addressing health issues and participating in health-related studies (52). Variations in ethnicity and marital status across gender may indicate wider societal and cultural dynamics, including differences in gender roles, family structures, and migration patterns, which affect lifestyle behaviors (49).

### 4.2 Dietary, physical, and social activities behaviors

Our analysis extends beyond confirming gender differences to reveal how distinct behavioral pathways lead to equivalent MedLife adherence. The analysis of the MedLife Index revealed several gender-related patterns in adherence to the MedLife, with both overlapping and distinct behaviors observed across its three core dimensions. While no significant differences emerged in the overall MedLife Index score, detailed analysis by block uncovered meaningful gender-specific variations. In Block 1 (Mediterranean Food Consumption), females scored significantly higher than males. This difference was driven by stronger adherence among women in 7 out of 15 dietary components, particularly in reducing red and processed meat intake, and in the consumption of vegetables, olive oil, and herbs. These findings are consistent with previous studies showing that women tend to prioritize long-term health outcomes and demonstrate greater nutritional awareness and restraint in food choices (53). Furthermore, hormonal and physiological differences may also play a role—estrogen, for example, positively influences lipid and glucose metabolism, aligning well with the plant-rich and unsaturated fat profile of the MedDiet (20, 54). In contrast, males reported greater adherence in fish/seafood, legumes, and sweets consumption, reflecting possible cultural preferences or gender-based differences in food availability or preparation habits (53, 55).

No significant gender differences were observed in Mediterranean dietary habits (block 2), indicating comparable general adherence to dietary routines such as moderation and meal timing. However, item-level analysis revealed divergent patterns. Females adhered more closely to recommendations for whole grain consumption, reducing sugar in beverages, and limiting snacking between meals, behaviors typically associated with weight control and preventive health (53, 56). These trends align with evidence that women are more likely to engage in health-protective dietary practices and report higher levels of dietary self-regulation (57, 58).

On the other hand, males showed better adherence in water or infusion consumption, wine intake, and salt limitation. Moderate wine consumption is culturally normalized for men in many Mediterranean societies and may be perceived as socially acceptable or even beneficial for cardiovascular health (59, 60). Higher adherence to hydration and salt limitation could reflect a stronger association with PA routines or awareness of cardiovascular risk (61, 62). This highlights the need for gender-sensitive dietary interventions, as males and females may respond differently to public health messaging depending on their motivations, lifestyles, and sociocultural context.

In Block 3 (PA, Rest, Social Habits, and Conviviality), males exhibited significantly higher scores, pointing to distinct lifestyle patterns between genders. This was particularly evident in components such as PA, collective sports, and social engagement. These trends were further supported by the IPAQ-SF results, which showed a higher prevalence of high-intensity PA among males compared to females, while low-intensity PA were more prevalent among females than males. Collectively, this contributed to higher total PA score in male than female considering that high intensity PA reflect higher MET values. These differences may stem from a combination of social, cultural, and practical factors. Men are often more involved in structured or vigorous activities, such as sports or physically demanding work, driven by societal norms that encourage physical competitiveness and outdoor engagement (63–65). Their participation in collective activities and higher self-reported intensity levels may also reflect greater opportunities or prioritization of leisure-time PA (66, 67).

In contrast, women may face greater barriers to sustained or intense activity due to competing demands such as caregiving and domestic responsibilities, which limit their discretionary time and energy for exercise (68–70). Consequently, even when women engage in PA, it is often of lower intensity or incorporated into daily routines (e.g., walking or household tasks) rather than structured sessions. Social engagement, another aspect of Block 3—is often linked to psychological well-being, and men’s greater participation in group-based activities may serve as both a physical and social health resource (71).

Despite these block-specific differences, the overall MedLife Index categories (low, medium, high adherence) did not differ significantly by gender, suggesting that men and women achieve similar levels of total MedLife adherence, albeit through different behavioral pathways. This reinforces the importance of designing gender-tailored interventions that account for varied motivators and constraints rather than adopting a one-size-fits-all model.

Turning to the perceived barriers, females reported significantly higher levels of perceived obstacles to adopting a MedLife. Detailed analysis showed that women were more likely to cite attitudinal barriers, lack of knowledge, and taste-related issues. These findings may reflect underlying disparities in access to health education, exposure to culturally relevant nutrition information, or a lack of supportive environments that facilitate healthy food choices (72, 73). Additionally, sensory preferences and food aversions are known to differ by gender, with women often more sensitive to taste, texture, or dietary restrictions imposed by family settings (74).

Conversely, men reported higher proportions of low motivation and health-related (medical) reasons, indicating that while women may face more environmental and knowledge-based barriers, men may be more affected by internal motivation or physical constraints. This aligns with previous findings showing that men are less likely to engage in health-promoting behaviors unless prompted by medical necessity or structured settings (64, 75).

### 4.3 Sleep parameters, insomnia severity and technology use behaviors

Our sleep findings provide new evidence about how gender differences in sleep patterns interact with dietary behaviors across different populations. The present analysis revealed several significant gender differences in sleep-related outcomes. Specifically, females exhibited longer sleep latency, lower sleep efficiency, poorer subjective sleep quality, longer sleep duration (mean value exceeding recommended duration), and greater insomnia severity (ISI scores) compared to males. These findings align with previous studies showing that women are more likely to report disrupted sleep patterns and symptoms of insomnia (76–78). The observed longer sleep latency and lower efficiency in females may be attributable to hormonal fluctuations (e.g., during menstrual cycles, pregnancy, or menopause) that may interfere with sleep regulation and stability (79). In addition, psychological and behavioral factors, such as a heightened stress response, a greater tendency for rumination, and increased exposure to caregiving responsibilities, may further delay sleep onset and contribute to night-time awakenings (80).

These sleep-based lifestyle disruptions, when combined with other social factors, such as increased caregiving, likely contribute to the significantly higher ISI scores observed in females, along with their higher proportion in the moderate-to-severe insomnia categories (81, 82). Interestingly, although females reported longer sleep duration, this may represent a compensatory behavior to offset the lower sleep quality and efficiency. Women often extend total sleep time in an effort to achieve restorative rest, despite experiencing more frequent awakenings and difficulty falling asleep (83, 84). This finding is consistent with the hypothesis that sleep quantity alone may not reflect true restfulness, especially in the presence of fragmented sleep architecture.

While significant differences were found in continuous scores for sleep quality and duration, no gender differences were observed in categorical classifications of sleep duration or quality (e.g., recommended duration or self-rated sleep quality categories). This discrepancy may suggest that while men and women differ in sleep patterns and subjective scores, they may be similarly distributed in terms of broader public health classifications.

The higher reported technology use among females may further contribute to impaired sleep outcomes. Excessive screen time—particularly before bedtime—has been associated with delayed sleep onset and reduced melatonin secretion, disproportionately affecting women who already exhibit greater sensitivity to sleep-disrupting stimuli (85–87).

### 4.4 Mental health: depression, anxiety, stress, and needs of support

Our mental health results strengthen evidence for gender-specific patterns while suggesting new directions for integrated interventions. The findings of this study revealed significant gender differences in psychological distress and perceived need for support. Females reported significantly higher levels of depression, anxiety, and stress, as measured by the DASS-21, with small-to-moderate effect sizes across all domains. These results are consistent with a broad body of literature indicating that women are more prone to internalizing disorders, likely due to a complex interplay of biological, psychological, and social factors (88, 89).

Hormonal fluctuations associated with the menstrual cycle, pregnancy, and menopause may contribute to increased vulnerability to mood dysregulation and heightened emotional reactivity in women (88). In addition, gender differences in emotional processing and coping styles may also play a role. Women tend to engage in emotion-focused coping strategies and are more likely to ruminate on negative experiences, which can exacerbate stress and depressive symptoms (89). In contrast, men are more likely to employ problem-focused coping and often underreport emotional difficulties, due in part to sociocultural norms that discourage emotional expression and promote stoicism (90). This may explain the significantly higher proportion of males in the “normal” range across all three psychological distress categories and their underrepresentation in mild to severe categories.

In terms of perceived need for support, females consistently reported significantly higher needs across all three domains: psycho-social, physical, and nutritional support. These findings reflect women’s greater inclination to recognize and express the need for help, particularly in response to emotional and physical strain (91). Women may also be more likely to seek social connection and professional assistance as part of their coping process, which is aligned with gender socialization patterns that emphasize interdependence, caregiving, and relational well-being (92).

On the other hand, males were significantly more likely to report no perceived need for support in any domain. This aligns with prior research suggesting that men often minimize their perceived need for help, particularly in health and emotional contexts, due to internalized beliefs about self-reliance and the stigma associated with vulnerability (93). This reluctance to acknowledge or seek support may contribute to unmet needs and could partially obscure underlying psychological struggles that are not outwardly expressed or captured by self-report measures.

### 4.5 Correlations

The interconnectedness of lifestyle factors revealed by our multinational sample provides robust evidence for holistic interventions. The MedLife Index demonstrates a significant correlation between adherence to the MedDiet and multiple health and lifestyle factors, such as PA, mental health, sleep quality, and social support.

The positive correlation observed between the MedLife Index and IPAQ-SF scores (*r* = 0.208) illustrates the relationship between dietary adherence and PA. This relationship indicates the broader concept of a MedLife, characterized by the integration of regular PA and a nutrient-rich diet. Engaging in PA improves energy balance, metabolic efficiency, and mental health, thereby complementing the anti-inflammatory and cardiovascular advantages associated with the MedDiet (94). Integrating bidomain interventions, combining PA with the MedDiet, may enhance health outcomes, particularly in populations with low PA levels (95).

The negative correlation between dietary adherence and insomnia severity suggests that the MedDiet may reduce insomnia severity through metabolic and neurological mechanisms. Its high fiber content helps stabilize blood sugar levels, while its anti-inflammatory components alleviate conditions that disrupt sleep (96). Furthermore, the MedDiet, characterized by a high intake of fruits, vegetables, whole grains, and omega-3 fatty acids, enhances sleep quality. These foods promote the synthesis of melatonin and serotonin, essential for regulating sleep–wake cycles and improving restorative sleep (97, 98). Promoting the MedDiet may improve sleep satisfaction and provide a non-pharmacological strategy for addressing sleep disturbances (99).

The positive correlation between the MedLife Index and social participation illustrates the significance of social connections in enhancing adherence to the MedDiet. Social networks influence dietary choices, promote the adoption of Mediterranean eating patterns, and offer emotional reinforcement (99). Individuals in supportive social environments are more likely to maintain adherence to diets, benefiting from shared meals, cultural practices, and collective motivation (99).

The positive correlation between the MedLife Index and life satisfaction, along with the inverse relationships observed between the MedLife Index and stress, anxiety, and depression, underscore the potential protective benefits of a MedDiet on mental health. This finding is consistent with research indicating that the anti-inflammatory and neuroprotective effects of the MedDiet can alleviate symptoms of psychological distress (100, 101). This diet high in antioxidants, polyphenols, and omega-3 fatty acids can mitigate oxidative stress and enhance cognitive function, which are essential for the management of anxiety, depression, and stress (101).

### 4.6 Potential inflation of significance due to large sample size

Although the large sample size in this study enhances statistical power and improves the potential generalizability of the findings, it may also contribute to the inflation of statistical significance. In large datasets, even minimal differences can yield statistically significant results, which do not necessarily reflect meaningful or practically relevant effects. As such, while the associations identified are noteworthy, they should be interpreted with caution. Further research, particularly studies employing targeted sampling or intervention-based designs, is needed to confirm these associations and evaluate their clinical or practical significance.

### 4.7 Strength and limitation

This study has several notable strengths, particularly its multinational scope and demographically diverse sample, which enhance the generalizability of findings across cultural contexts. The use of validated tools to assess demographic, behavioral, and psychosocial factors provides a comprehensive overview of participants’ lifestyle characteristics and their associations with MedDiet adherence. Additionally, gender-specific analyses offer nuanced insights into differences in dietary and lifestyle behaviors between men and women, contributing to a more differentiated understanding of adherence dynamics.

However, several limitations must be acknowledged. The cross-sectional design inherently limits the ability to draw causal inferences between adherence patterns and health outcomes. The study also faces the potential for self-selection bias, as participation was voluntary and conducted online, which may have attracted younger, more educated, urban, and health-conscious individuals—while underrepresenting older adults and those in rural settings. Social desirability and recall biases may have been introduced through the reliance on self-reported data, even when collected using validated instruments such as the MedLife Index and IPAQ-SF. Unmeasured confounders, including socioeconomic disparities, cultural influences, or region-specific food environments, may also have influenced the results and reduced precision in subgroup comparisons.

Furthermore, cultural biases in survey responses across different countries should be considered. Despite using validated instruments, cultural differences in dietary practices, health perceptions, and response styles may have influenced participants’ answers. For example, varying interpretations of food-related behaviors and lifestyle choices across countries could impact the accuracy of self-reported data. Additionally, cultural norms around health and nutrition may have led to different levels of social desirability bias, particularly in more health-conscious populations. Regional differences in food availability, traditional eating habits, and lifestyle factors may have introduced further variability in the results, potentially reducing the comparability of data across countries.

While we conducted gender-specific analyses to explore key differences between men and women, the unequal gender distribution and potential selection bias may have affected the robustness of subgroup comparisons. To address this, we applied proportional analytical approaches and statistical techniques, such as Z-tests for two proportions, to detect gender-related differences while accounting for unequal sample sizes.

Future studies should aim for more balanced gender representation, adopt stratified sampling methods, incorporate objective data collection (e.g., biomarkers or wearable devices), and employ longitudinal or interventional designs to strengthen causal interpretation and validate these findings in more representative populations.

## 5 Conclusion

This study highlights the complex and gender-specific patterns underlying adherence to the Mediterranean lifestyle. While females demonstrated greater compliance with Mediterranean dietary recommendations, males reported higher engagement in PA, rest, and social participation. Furthermore, the MedLife Index was positively associated with favorable outcomes such as PA levels, life satisfaction, and social engagement, and negatively associated with insomnia severity, stress, anxiety, and depressive symptoms.

These findings emphasize the need for gender-sensitive and behaviorally targeted interventions that consider the distinct motivations, barriers, and cultural contexts influencing lifestyle choices. Tailoring public health strategies to these differences may enhance the adoption and sustainability of the Mediterranean lifestyle and ultimately improve population health outcomes.

## 6 Practical applications

The findings of this study carry important implications for public health strategies aimed at promoting adherence to the MedLife, particularly in diverse populations. Although overall adherence to the MedLife Index was comparable between sexs, the behavioral components contributing to these scores varied significantly, indicating the need for gender-tailored interventions.

For instance, women demonstrated higher adherence to dietary components of the Mediterranean lifestyle, yet lower engagement in PA, greater psychological distress, and higher perceived barriers to healthy behaviors. Accordingly, initiatives targeting women should emphasize flexible, time-efficient, and culturally appropriate PA options, such as group fitness classes, outdoor walking groups, or family-oriented activities that can be integrated into daily routines. At the same time, interventions should address the attitudinal and structural barriers reported by women, including taste preferences, time constraints, and lack of nutrition knowledge. Empowerment through community-based cooking workshops, peer-led support groups, and the use of digital tools (e.g., mobile apps for meal planning or stress management) could enhance self-efficacy and engagement.

In contrast, men reported greater PA levels and higher scores in social participation and rest-related behaviors but showed lower adherence to dietary recommendations. For this group, strategies should focus on improving nutrition behaviors through targeted education on key dietary components, such as reducing sugar intake and increasing whole grain consumption, while also promoting awareness about hydration and salt moderation. Practical approaches might include structured nutrition counseling, goal-setting interventions, or hands-on cooking sessions that align with men’s reported preferences for performance-oriented or outcome-driven programs. Furthermore, addressing motivational and health-related barriers, including medical concerns and low perceived risk, may enhance dietary engagement among men.

Importantly, the MedLife Index was positively associated with PA, life satisfaction, and social participation, and negatively associated with insomnia, stress, anxiety, and depression. This highlights the potential of comprehensive lifestyle interventions that not only target diet and activity but also integrate mental health support and sleep hygiene strategies. For women in particular, interventions should address psychosocial stressors such as caregiving burden and emotional regulation difficulties, possibly through mindfulness training, peer support networks, or flexible access to mental health services. For men, outreach efforts may need to tackle help-seeking stigma and encourage early engagement with psychological support, even in the absence of severe symptoms.

The gender-specific disparities in sleep quality, latency, efficiency, and insomnia severity also point to the need for tailored sleep promotion initiatives. For women, this may include education on the impact of hormonal cycles, stress management tools, and limiting technology use before bedtime. For men, reinforcing existing protective behaviors and encouraging consistent sleep routines can help maintain optimal sleep health.

Finally, the significantly higher perceived need for psychosocial, physical, and nutritional support among women, alongside the tendency for men to underreport such needs, further supports the use of personalized health strategies. Public health policies should promote inclusive and culturally sensitive education campaigns, ensure equitable access to support services, and leverage digital health solutions to deliver timely, scalable, and adaptable interventions that meet the unique needs of each gender.

Together, these practical insights underscore the necessity of multi-dimensional, gender-sensitive, and context-specific interventions to sustainably promote the Mediterranean lifestyle and its associated health benefits across diverse populations.

## Data Availability

The datasets generated and analyzed during the current study are not publicly available at this time as further analyses are ongoing, and additional publications based on these data are in preparation. Data may be made available upon reasonable request to the corresponding author once all planned analyses and publications are completed.
