# Correction: Gender-specific insights into adherence to Mediterranean diet and lifestyle: analysis of 4,000 responses from the MEDIET4ALL project

**DOI:** 10.3389/fnut.2025.1692429

**Published:** 2025-09-30

**Authors:** Mohamed Ali Boujelbane, Achraf Ammar, Atef Salem, Mohamed Kerkeni, Khaled Trabelsi, Bassem Bouaziz, Liwa Masmoudi, Juliane Heydenreich, Christiana Schallhorn, Gabriel Müller, Ayse Merve Uyar, Hadeel Ali Ghazzawi, Adam Tawfiq Amawi, Bekir Erhan Orhan, Giuseppe Grosso, Osama Abdelkarim, Tarak Driss, Kais El Abed, Piotr Zmijewski, Nasreddine Benbettaieb, Clément Poulain, Laura Reyes, Amparo Gamero, Marta Cuenca-Ortolá, Nicola Francesca, Concetta Maria Messina, Björn Lorenzen, Stefania Filice, Aadil Bajoub, El-Mehdi Ajal, El Amine Ajal, Majdouline Obtel, Sadjia Lahiani, Taha Khaldi, Nafaa Souissi, Omar Boukhris, Haitham Jahrami, Waqar Husain, Walid Mahdi, Hamdi Chtourou, Wolfgang I. Schöllhorn

**Affiliations:** ^1^Department of Training and Movement Science, Institute of Sport Science, Johannes Gutenberg-University Mainz, Mainz, Germany; ^2^High Institute of Sport and Physical Education of Sfax, University of Sfax, Sfax, Tunisia; ^3^Research Laboratory, Molecular Bases of Human Pathology, LR19ES13, Faculty of Medicine of Sfax, University of Sfax, Sfax, Tunisia; ^4^Department of Movement Sciences and Sports Training, School of Sport Science, The University of Jordan, Amman, Jordan; ^5^Research Laboratory Education, Motricity, Sport and Health, EM2S, LR19JS01, High Institute of Sport and Physical Education of Sfax, University of Sfax, Sfax, Tunisia; ^6^Multimedia InfoRmation systems and Advanced Computing Laboratory (MIRACL), University of Sfax, Sfax, Tunisia; ^7^Higher Institute of Computer Science and Multimedia of Sfax (ISIMS), University of Sfax, Sfax, Tunisia; ^8^Faculty of Sports Sciences, Department of Experimental Sports Nutrition, Leipzig University, Leipzig, Germany; ^9^Department of Sports Economics, Sociology and History, Institute of Sport Science, Johannes Gutenberg-University Mainz, Mainz, Germany; ^10^Department of Nutrition and Food Technology, School of Agriculture, The University of Jordan, Amman, Jordan; ^11^Faculty of Sports Sciences, Istanbul Aydin University, Istanbul, Türkiye; ^12^Department of Biomedical and Biotechnological Sciences, University of Catania, Catania, Italy; ^13^Faculty of Sport Sciences, Assiut University, Assiut, Egypt; ^14^ESLSCA University Egypt, Giza, Egypt; ^15^Interdisciplinary Laboratory in Neurosciences, Physiology and Psychology Physical Activity, Health and Learning (LINP2), UFR STAPS, Paris Nanterre University, Nanterre, France; ^16^Department of Biomedical Sciences, Jozef Pilsudski University of Physical Education in Warsaw, Warsaw, Poland; ^17^Department BioEngineering, Institut Universitaire de Technologie IUT-Dijon-Auexrre-Nevers, University Burgundy Europe, Dijon, France; ^18^Joint Research Unit UMR PAM-PCAV (Physical-Chemistry of Food and Wine Laboratory), Université Bourgogne Europe/Institut AgroDijon/INRAE, Dijon, France; ^19^Vitagora Innovation Cluster, Dijon, France; ^20^Department of Preventive Medicine and Public Health, Food Science, Toxicology and Forensic Medicine, Faculty of Pharmacy & Food Sciences, University of Valencia, Valencia, Spain; ^21^Department of Agricultural Food and Forest Sciences, University of Palermo, Palermo, Italy; ^22^Laboratory of Marine Biochemistry and Ecotoxicology, Department of Earth and Marine Sciences DiSTeM, University of Palermo, Trapani, Italy; ^23^Microtarians Academy, Luxembourg, Luxembourg; ^24^Laboratory of Food and Food By-Products Chemistry and Processing Technology, National School of Agriculture in Meknès, Meknès, Morocco; ^25^Laboratory of Social Medicine, Faculty of Medicine and Pharmacy of Rabat, Department of Epidemiology and Public Health, Mohammed V University, Rabat, Morocco; ^26^UPR of Pharmacognosy, Faculty of Medicine and Pharmacy of Rabat, Mohammed V University, Rabat, Morocco; ^27^VALCORE Laboratory, Faculty of Science, Department of Biology, University of M'hamed Bougara Boumerdes, Boumerdes, Algeria; ^28^Biotechnology Research Center (C.R.Bt), Constantine, Algeria; ^29^SIESTA Research Group, School of Allied Health, Human Services and Sport, La Trobe University, Melbourne, VIC, Australia; ^30^Sport, Performance, and Nutrition Research Group, School of Allied Health, Human Services and Sport, La Trobe University, Melbourne, VIC, Australia; ^31^Government Hospitals, Manama, Bahrain; ^32^Department of Psychiatry, College of Medicine and Medical Sciences, Arabian Gulf University, Manama, Bahrain; ^33^Department of Humanities, COMSATS University Islamabad, Islamabad, Pakistan

**Keywords:** Mediterranean diet, gender differences, MedLife index, physical activity, mental health

In the published article, there was a mistake in Figure 1. Figure 1 illustrates the geographical distribution and sample sizes of study participants from selected Mediterranean and non-Mediterranean countries. However, the figure mistakenly contained an incorrect representation of the Moroccan border. The corrected Figure 1 and its caption appear below.

**Figure 1:**
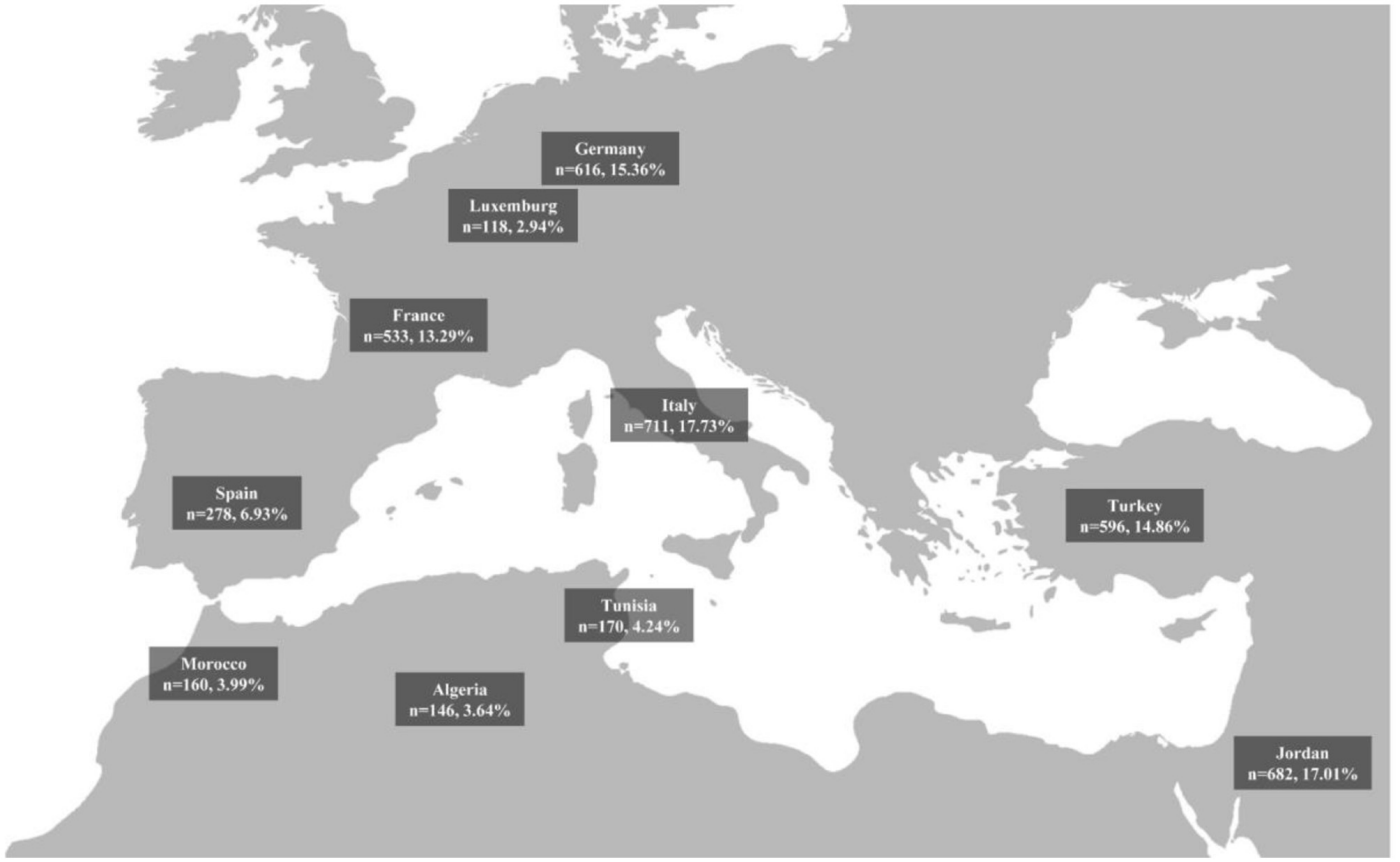
Geographical distribution and sample sizes of study participants from selected mediterranean and non-mediterranean countries.

The original article has been updated.

